# When synchronous mucinous metaplasia and neoplasia of the female genital tract and peutz-jeghers syndrome meet: a case report and literature reviews

**DOI:** 10.1186/s12905-024-03184-y

**Published:** 2024-06-28

**Authors:** Yue Zhou, Xinyi Wang, Yang Li, Weiru Zhang, Xiaoxuan Xu, Yingxin Pang, Peishu Liu

**Affiliations:** 1 College of Medicine, Cheeloo College of Medicine, Shandong University, 44 Wenhua Xi Road, Jinan, Shandong People’s Republic of China; 2 Department of Obstetrics and Gynecology, Qilu Hospital of Shandong University, 107 Wenhua Xi Road, Jinan, Shandong People’s Republic of China; 3 Shandong Engineering Laboratory for Urogynecology, Qilu Hospital of Shandong University, Jinan, Shandong People’s Republic of China

**Keywords:** Peutz-Jeghers syndrome, Adenocarcinoma, STK11, Mucinous metaplasia and neoplasia, Female genital tract

## Abstract

**Background:**

Peutz-Jeghers syndrome (PJS) is characterized by the presence of hamartomatous polyps in the gastrointestinal tract and mucocutaneous pigmentation on the lips, oral mucosa, nose, fingers, and toes. Synchronous mucinous metaplasia and neoplasia of the female genital tract (SMMN-FGT) refers to the occurrence of multifocal mucinous lesions in at least two sites, including the cervix, uterus, fallopian tubes, and ovaries, in the female genital tract. SMMN-FGT and PJS are rare diseases with a very low incidence, especially when occurring simultaneously.

**Case presentation:**

We report a case in which a woman with a large mass on the left ovary underwent a gynecological surgery and was diagnosed with cervical gastric-type adenocarcinoma and mucinous lesions in the endometrium, bilateral fallopian tubes, and ovary, i.e., SMMN-FGT, by postoperative paraffin pathology. The patient sought medical attention for abdominal distension and enlargement. A gynecological ultrasound revealed a multilocular cystic mass in the pelvis, while serum tumor markers were within normal limits, with mildly elevated carbohydrate antigen 199 and carbohydrate antigen 125 levels. Cervical thin-prep cytology test result was negative. The patient had a family history of PJS with black spots on her skin and mucous membranes since the age of 8 years. She underwent multiple partial small bowel resections and gastrointestinal polypectomy owing to intestinal obstruction and intussusception. She underwent left adnexectomy, hysterectomy, right salpingectomy, greater omental resection, appendectomy and right ovary biopsy, and received six courses of adjuvant chemotherapy with Lopressor plus Carboplatin. Genetic testing revealed a heterozygous serine threonine kinase 11 germline mutation and there were no signs of recurrence during the 18-month follow-up period after treatment.

**Conclusions:**

This is a rare case in which PJS was complicated by SMMN-FGT. Owing to its extreme rarity, there are no guidelines, but reported cases appear to indicate a poor prognosis. We retrospectively reviewed all cases of collisions between PJS and SMMN-FGT and explored the clinical features, pathological characteristics, diagnosis, treatment methods, and prognosis when the two diseases coexisted. The aim is to deepen the clinicians’ understanding of this disease for early detection, diagnosis and treatment.

**Supplementary Information:**

The online version contains supplementary material available at 10.1186/s12905-024-03184-y.

## Background

Peutz-Jeghers syndrome (PJS) was first reported by Peutz in 1921 and later by Jeghers et al. with a prevalence of approximately 1/50,000-200,000 [1].It is characterized by hamartomatous polyps in the gastrointestinal tract and mucocutaneous pigmentation on the lips, oral mucosa, nose, fingers, and toes. Evidence suggests that patients with PJS have an increased risk of developing cancer, including gastrointestinal and extraintestinal cancers [2]. Female patients with PJS tend to have mucinous lesions in their genital organs, ranging from cystic or hyperplastic benign lesions to borderline neoplasms and to malignant tumors. Synchronous mucinous metaplasia and neoplasia of the female genital tract (SMMN-FGT) [3] refers to the occurrence of multifocal mucinous lesions in at least two sites in the female genital tract. It is a rare disease, and the diagnostic standards, treatment options, and follow-up criteria have not yet been standardized. Low morbidity, lack of specific symptoms, and challenges in preoperative diagnosis can lead to frequent clinical misdiagnosis or missed diagnosis, resulting in delayed treatment. Patients with PJS have a higher lifetime risk of cancer, whereas those with SMMN-FGT have a poor prognosis. SMMN-FGT and PJS are both rare diseases with very low incidence, especially they occur simultaneously. However, the symptoms of PJS may provide evidence of SMMN-FGT. Herein, we report a case of a woman with a long history of PJS who was diagnosed with SMMN-FGT in our hospital. We also reviewed all reported cases in which the two diseases coexisted to alert clinicians to the importance of early detection, diagnosis, and treatment of malignant tumors in these patients. Additionally, we stress the importance of cancer risk surveillance in individuals with PJS.

## Case presentation

A 38-year-old woman visited our hospital on 24 October 2022 for “abdominal distension and gradual enlargement of the lower abdomen for more than 3 months” and was diagnosed with SMMN-FGT after gynecological surgery. Gynecological examinations and ancillary tests revealed no specific changes. Uterine body palpation was unsatisfactory, and a large cystic mass reaching the umbilicus was palpable in the adnexal area. The cervix was smooth, with a small amount of transparent fluid flowing out. Ultrasound at our hospital revealed a multilocular cystic mass in the pelvis with multiple dense partitions inside. Blood flow signals were found above the partitions, and the cyst was hypoechoic and filled with fine and dense spots. The cervical thin-prep cytology test (TCT) was negative, and serum tumor markers were normal with carbohydrate antigen 199 (CA199) and carbohydrate antigen 125 (CA125) mildly elevated. No other abnormal radiological or laboratory results were observed. In summary, the possible diagnosis of the female patient was an ovarian tumor.

**Fig. 1:**
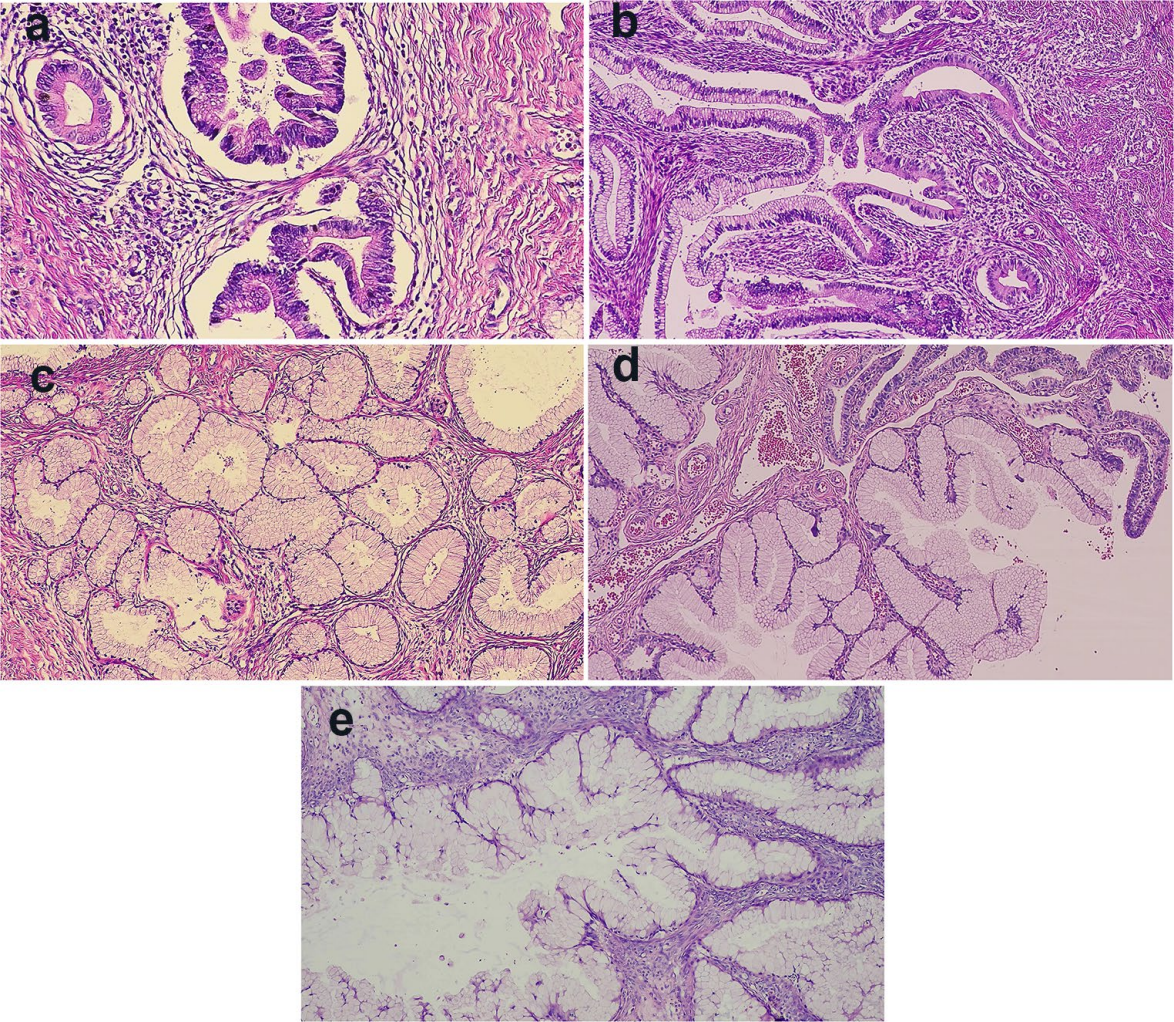
Pathology findings after surgery (H-E stain), (**A**) gastric-type adenocarcinoma of cervical (×100), (**B**) cervical lobular mucinous gland hyperplasia (×100), (**C**) proliferation of mucinous glands in endometrium (×100), (**D**) mucinous gland metaplasia and mild atypia proliferation of right fallopian tube (×100), (**E**)mucinous cystic tumor of left ovary (×100)

Considering the possibility of a malignant tumor, the patient underwent laparoscopic exploration and subsequent laparotomy on 28 October 2022 and was finally diagnosed with cervical gastric-type adenocarcinoma. During laparoscopic exploration, a large amount of yellowish viscous fluid and a large mass on the left ovary with a diameter of 20 cm were observed, while the uterus, right ovary and other organs were normal to the naked eye. Therefore, left adnexectomy and right ovary biopsy were performed firstly. Intraoperative frozen section pathology revealed borderline mucinous tumors in the left ovary and cortical stromal hyperplasia in the right ovary. Hysterectomy, right salpingectomy, greater omental resection, and appendectomy were performed subsequently. Postoperative pathology showed gastric-type adenocarcinoma of the cervix, with a lesion involving approximately 80% of the entire uterine wall and a depth of approximately 10 mm, proliferation of mucinous glands in the endometrium, borderline mucinous cystic tumor of the left adnexa, mucinous gland metaplasia, and mild atypical proliferation of the right fallopian tube (Fig. 1). There was proliferation of mucinous glands in the cervix, endometrium, bilateral fallopian tubes, and left ovary, with some glandular dysplasia and tumor formation, suggesting concurrent mucinous metaplasia and tumors, i.e., SMMN-FGT.

Because of the young age and benign result of the right ovary biopsy, supplemental surgery was not conducted, and postoperative adjuvant chemotherapy was administered instead. On 15 November 2022 the patient underwent the first course of chemotherapy with a regimen of Paclitaxel Liposome for Injection plus Carboplatin. Six courses of chemotherapy were administered with the last course on 17 April 2023, and no signs of recurrence were observed during the 18-month follow-up period after surgery.

It is worth noting that this patient had black spots on her skin and mucous membranes since the age of 8 years, mainly distributed on the face, lips, fingers, and toes; these spots partially diminished after she gave birth to her daughter in 2008. In 2001, she underwent a partial small bowel resection owing to “intestinal obstruction” at a local hospital and was diagnosed with PJS based on postoperative pathology. She underwent multiple surgeries to remove the gastrointestinal polyps and a part of the small intestine owing to recurrence. Genetic testing revealed a heterozygous type serine threonine kinase 11 (STK11) germline mutation. The patient’s father died of liver cancer associated with PJS. Her mother is healthy, while her younger brother had a history of PJS and has undergone several surgeries So far, no extraintestinal cancers have been found. Her daughter is currently 15 years old and has not exhibited any black spots or other symptoms.

## Discussion and conclusions

SMMN-FGT combined with PJS is extremely rare, with only 11 cases reported [4–13] to our knowledge (Table 1). The patients were aged between 30 and 54 years, with one aged 75 years. Its clinical presentation is non-specific, with most patients complaining of pelvic masses, abnormal vaginal bleeding, increased vaginal discharge, or abdominal discomfort. The patient in this study was 38 years old, presented with abdominal distension and was diagnosed with a pelvic mass on imaging. However, upon reviewing the patient’s medical history, it was found that she had experienced thin, transparent vaginal discharge for more than a year, which she believed to be normal vaginal secretions and did not pay much attention to. Finally, the possibility of cervical tumors was overlooked because of the negative result of cervical TCT result. Auxiliary examinations, including imaging studies and serum tumor markers, show no obvious specificity, and ultrasonography often suggests polycystic lesions in the affected areas. Tumor markers are usually normal, however, when accompanied by gastric-type adenocarcinoma, an increase in CA199 is common, while CA125 is often normal or slightly elevated.

**Table 1:** Reported cases of co-occurrence of SMMN-FGT and PJS

Case No.	Age (y)	STK11	Presentation	Pathology	Primary surgery	Adjuvant therapy	Metastasis /Recurrence	Follow-up	Author	Date
1	36	NA	abdominal pain	intestinal-like metaplasia of fallopian tube epithelium, SCTAT of ovary, metastasizing adenocarcinoma of cervix	radical hysterectomy and BSO	NA	metastasis, 3 years	DOD, 19mo after metastasis	COSTA J [4]	1976
2	54	NA	NA	SCTAT of ovary, MDA of cervix, metaplasia of fallopian tubes	NA	NA	NA	NA	BERGER G [5]	1981
3	33	NA	menometrorrhagia and an adnexal mass	SCTAT, OMC, MDA of cervix, metaplasia of fallopian tubes	total hysterectomy and RSO	CT	recurrence, 2 mo	DOD, 7 mo after recurrence	Chen [6]	1986
4	40	NA	pelvic mass	SCTAT of ovary, cervical adenoma malignum	total hysterectomy and BSO	CT, RT after metastasis	metastasis, 5 years	DOD, 4 mo after metastasis	Podezaski E [7]	1991
5	42	NA	persistent leukorrhea	invasive mucinous carcinoma of cervix, SCTAT of ovary, focal adenocarcinoma of endometrium	total hysterectomy and BSO	NA	NA	dead of unknown reason, 54 mo	SEIDMAN JD [8]	1994
6	42	NA	abdominal pain, left adnexal mass	MDA of cervix, mucosal metaplasia of fallopian tubes, borderline malignancy of R ovary	total hysterectomy and LSO, bilateral sampling of external iliac lymph nodes and ileal resection ^a^	No	NA	NED, 21 mo	MANGILI G [9]	2004
7	53	NA	NA	pyloric gland metaplasia and differentiation of uterine cervix, fallopian tubes and ovary	total hysterectomy and BSO	NA	NA	NA	Kato N [10]	2011
8	30	Yes	fallopian tube mass	mucinous hyperplasia of fallopian tube and ovary	NA	NA	recurrence, 3 mo	DOD, 43 mo	Bennett JA [11]	2021
9	33	NA	abnormal vaginal bleeding	LEGH of cervix, multifocal gastric-type MCM of endometrium and fallopian tubes	total hysterectomy and BS	No	No	NED, 43mo	Chen [12]	2022
10	37	NA	abnormal vaginal bleeding	LEGH of cervix and endometrium	total hysterectomy and BS	No	No	NED, 29mo	Chen [12]	2022
11	75	NA	elevation of the CA199	malignant transformation of R fallopian tube, mucinous hyperplasia of L fallopian tube and R ovary	total hysterectomy and BSO	CT	metastasis, 2 years	DOD, several mo	Bronte Anaut M [13]	2023

*Abbreviation* R: right; L: left; DOD: dead of disease; NA: not available; NED: no evidence of disease; RT: radiotherapy; CT: chemotherapy; SCTAT: Sex cord tumor with annular tubules; MDA: minimal deviation adenocarcinoma; BSO: bilateral salpingo-oophorectomy; RSO: right salpingo-oophorectomy; LSO: left salpingo-oophorectomy; BS: bilateral salpingectomy; LEGH: lobular endocervical glandular hyperplasia; MCM: mucous cell metaplasia; OMC: ovarian mucinous cystadenoma. “^a^” indicates that the patient was underwent a right salpingo-oophorectomy because of a serous-papillary cystoadenoma 6 years ago

Owing to the rarity of SMMN-FGT, there is currently no internationally recognized standard treatment. Most reported cases underwent total hysterectomy and bilateral salpingo-oophorectomy. Some studies [14] suggest that ovarian preservation may not be recommended for gastric-type adenocarcinoma patients and consider omentectomy as part of surgical treatment. Regarding adjuvant therapy, different physicians have varying opinions on whether and what kind of adjuvant therapy should be administered postoperatively. As shown in Table 1, some patients received adjuvant chemotherapy postoperatively, whereas others with benign lesions only underwent close follow-up without postoperative adjuvant therapy. Radiotherapy has been used in patients with metastasis. However, the role of adjuvant therapy could not be assessed because of lack of information. In our case, the patient underwent left adnexectomy, hysterectomy, right salpingo-oophorectomy, greater omental resection and appendectomy. In addition, the right ovary was preserved because intraoperative frozen pathology suggested that no tumor was detected in the right ovary. However, this may have been inadequately treated. Finally, the patient received six courses of postoperative adjuvant chemotherapy with Lipiodol plus Carboplatin. Diagnosis of SMMN-FGT relies on postoperative pathology with the presence of mucinous lesions in at least two sites in the female genital tract. Of the reported cases, nine involved the cervix; nine ovaries; eight fallopian tubes; and three endometrium, with pathological types ranging from mucous hyperplasia and metaplasia to carcinoma. In our case, mucinous lesions involved four sites in the reproductive tract: the cervix, endometrium, fallopian tubes, and left ovary.

The disease prognosis depends on the grade of the most severe lesion, with gastric-type adenocarcinoma having the worst prognosis [15]. Of the previously reported cases, two were lost to follow-up, three showed no signs of disease recurrence, and the remaining cases died of disease recurrence, metastasis, or other causes, with a follow-up time of no more than 5 years, suggesting a poor prognosis. Of the three surviving patients, two (case 9 and 10) had bilateral ovaries preserved owing to benign intraoperative pathology. These patients were young, and they were followed up without adjuvant treatment because of benign lesions. As for the last follow-up, conducted at 43 and 29 months, respectively, neither patient showed signs of recurrence. However, in view of the possibility of malignancy, close follow-up is recommended, and prophylactic resection of both ovaries should be considered after menopause. One malignant case was followed up for 21 months without signs of recurrence, which may have been related to extensive surgery (case 6, with partial resection of the bowel). The patient in our case is currently being followed up for 18 months, with no signs of recurrence. This may be attributed to postoperative adjuvant chemotherapy; however, as one ovary was preserved, close follow-up is required.

The pathogenesis of SMMN-FGT is unknown, and the histological changes are closely related to pyloric gland metaplasia and exhibits a series of morphological features ranging from metaplasia to invasive mucinous adenocarcinoma with a complex mechanism. Morphological features of SMMN-FGT include simple gastric-type mucinous metaplasia, papillary growth, atypical papillary growth, microinvasive adenocarcinoma, gastric-type adenocarcinoma, mucinous metaplasia of the fallopian tube, ovarian mucinous cystadenoma, and borderline or mucinous ovarian carcinoma. Therefore, we hypothesize that SMMN-FGT is a group of diseases consisting of benign lesions that progressively become malignant over time.

PJS is an autosomal dominant disorder caused by mutations in the liver kinase B1 (LKB1) gene, also known as STK11 gene, which functions as a tumor suppressor gene and is located on chromosome 19p13.3 [16]. Mutations in this gene can dysregulate various signaling pathways such as mTOR, Wnt/β-catenin, or TCF-4, leading to the development of PJS, but the exact mechanism remains unclear. SMMN-FGT may be associated with genes related to the PJS, such as STK11. Jennifer reported [11] 22 examples of novel adnexal tumors associated with PJS in nearly 50% of the cases that harbored STK11 alterations. In published cases, the STK11 altered status remains unknown owing to age and technical limitations, but the presence of the STK11 mutation was confirmed in our case, which suggests that SMMN-FGT is associated with the STK11 mutation. However, the histogenesis of SMMN-FGT remains unclear, and further studies, especially transcriptomic, epigenetic, and proteomic analyses, are warranted.

Patients with PJS have a higher risk of developing malignant tumors, therefore, early detection and regular monitoring of high-risk patients are crucial. No clear guidelines currently exist for the surveillance of high-risk cancers of the female genital tract secondary to the PJS. However, relevant study [17] have indicated that transvaginal ultrasound, serum CA125 test, pelvic examination and cervical smear should be performed annually, beginning at 18 years of age. Simultaneously, attention should be paid to menstruation and leucorrhea. In case of persistent abnormal uterine bleeding or vaginal discharge, even if the cervical TCT is negative, a biopsy should be performed, if necessary, for early diagnosis. In addition, owing to its rare onset and challenging diagnosis, gynecologists, gastroenterologists, dermatologists, urologists, and pathologists need to be more vigilant in their clinical practice.

Patient education, including, regular follow-ups, genetic counseling, early screening, and predictive diagnosis of their direct relatives, is particularly important. Patients should be informed of the high susceptibility of PJS to cancer and urged to follow-up regularly as prescribed by their doctor, note any symptoms of discomfort, and seek medical attention in a timely manner. Completion of genetic testing is recommended to guide follow-up and treatment and to provide a basis for monitoring the disease in relatives. Considering the genetic predisposition, early screening and predictive diagnosis of direct relatives are crucial. It is recommended that the patient’s 15-year-old daughter undergo regular medical examinations early on. The American College of Gastroenterology recommends regularly monitoring of patients with PJS from a young age, with upper gastrointestinal and small bowel series examinations starting at the age of 8 years and colonoscopy every 3 years thereafter [18]. Regular pelvic imaging should be performed in adulthood, with attention to menstrual status and abnormal vaginal discharge. The appearance of mucocutaneous pigmentation should be closely monitored, and, genetic testing should be performed to clarify the presence of STK11 mutations.

In conclusion, SMMN-FGT refers to multifocal mucinous lesions occurring simultaneously in the female genital tract. Its onset is rare, especially when complicated with PJS. The occurrence of SMMN-FGT may be associated with mutations in STK11, a gene associated with PJS. The clinical presentation is nonspecific, and diagnosis relies on pathological examination, which is often a diagnostic challenge for pathologists because of the extensive nature of the lesions. Surgical resection is the treatment of choice; however, the efficacy of postoperative adjuvant therapy is unknown with poor prognosis. Therefore, the early detection and regular medical surveillance of high-risk patients are crucial. Patients with PJS are advised to undergo regular medical check-ups for early detection of other malignancies, and their immediate family members must be screened regularly. Gynecologists should consider the possibility of SMMN-FGT when observing female patients with PJS and strive for early detection, diagnosis, and treatment. Gastroenterologists, dermatologists and urologists should be more alert to the symptoms of PJS in their clinical work.

### Electronic supplementary material

Below is the link to the electronic supplementary material.

Supplementary Material 1

## Data Availability

Data sharing is not applicable to this article as no datasets were generated or analysed during the current study.

## Abbreviations

PJS: Peutz-Jeghers syndrome
SMMN-FGT: Synchronous mucinous metaplasia and neoplasia of the female genital tract
CA125: Carbohydrate antigen 125
CA199: Carbohydrate antigen 199
STK11: Serine threonine kinase 11
LKB1: Liver kinase B1
TCT: Thin-prep cytology test

## References

[1] Jeghers H, Mc KV, Katz KH (1949). Generalized intestinal polyposis and melanin spots of the oral mucosa, lips and digits; a syndrome of diagnostic significance. N Engl J Med.

[2] Chen HY, Jin XW, Li BR, Zhu M, Li J, Mao GP, Zhang YF, Ning SB (2017). Cancer risk in patients with Peutz-Jeghers syndrome: a retrospective cohort study of 336 cases. Tumour Biol.

[3] Mikami Y, Kiyokawa T, Sasajima Y, Teramoto N, Wakasa T, Wakasa K, Hata S (2009). Reappraisal of synchronous and multifocal mucinous lesions of the female genital tract: a close association with gastric metaplasia. Histopathology.

[4] Costa J (1977). Peutz-Jeghers syndrome: case presentation. Obstet Gynecol.

[5] Berger G, Frappart L, Berger F, Seffert P, Serain F, Lamerant P, Feroldi J (1981). [Sex cord tumor with annular tubules, mucinous metaplasia of tubal epithelium, cystic and mucinous hyperplasia of the endocervix, and the Peutz-Jeghers syndrome (author’s transl)]. Arch Anat Cytol Pathol.

[6] Chen KT (1986). Female genital tract tumors in Peutz-Jeghers syndrome. Hum Pathol.

[7] Podczaski E, Kaminski PF, Pees RC, Singapuri K, Sorosky JI (1991). Peutz-Jeghers syndrome with ovarian sex cord tumor with annular tubules and cervical adenoma malignum. Gynecol Oncol.

[8] Seidman JD (1994). Mucinous lesions of the fallopian tube. A report of seven cases. Am J Surg Pathol.

[9] Mangili G, Taccagni G, Garavaglia E, Carnelli M, Montoli S (2004). An unusual admixture of neoplastic and metaplastic lesions of the female genital tract in the Peutz-Jeghers Syndrome. Gynecol Oncol.

[10] Kato N, Sugawara M, Maeda K, Hosoya N, Motoyama T (2011). Pyloric gland metaplasia/differentiation in multiple organ systems in a patient with Peutz-Jegher’s syndrome. Pathol Int.

[11] Bennett JA, Young RH, Howitt BE, Croce S, Wanjari P, Zhen C, Da Cruz Paula A, Meserve E, Schoolmeester JK, Westbom-Fremer S (2021). A distinctive Adnexal (usually Paratubal) Neoplasm often Associated with Peutz-Jeghers Syndrome and characterized by STK11 alterations (STK11 Adnexal Tumor): a report of 22 cases. Am J Surg Pathol.

[12] 陈默 李佳佳 (2022). 陶祥, 朱辰琪, 董戌晖, 尧良清, 李勤: 女性生殖道同期发生的黏液性上皮化生和肿瘤14例分析. 复旦学报(医学版).

[13] Bronte Anaut M, Arredondo Montero J, Fernández Seara MP, Guarch Troyas R (2023). Gastric-phenotype Mucinous Carcinoma of the fallopian tube with secondary ovarian involvement in a woman with Peutz-Jeghers syndrome: a Case Report. Int J Surg Pathol.

[14] Pirog EC, Park KJ, Kiyokawa T, Zhang X, Chen W, Jenkins D, Quint W (2019). Gastric-type adenocarcinoma of the Cervix: Tumor with wide range of histologic appearances. Adv Anat Pathol.

[15] Karamurzin YS, Kiyokawa T, Parkash V, Jotwani AR, Patel P, Pike MC, Soslow RA, Park KJ (2015). Gastric-type endocervical adenocarcinoma: an aggressive Tumor with unusual metastatic patterns and poor prognosis. Am J Surg Pathol.

[16] Hemminki A, Markie D, Tomlinson I, Avizienyte E, Roth S, Loukola A, Bignell G, Warren W, Aminoff M, Höglund P (1998). A serine/threonine kinase gene defective in Peutz-Jeghers syndrome. Nature.

[17] Piombino C, Cortesi L, Lambertini M, Punie K, Grandi G, Toss A (2020). Secondary Prevention in Hereditary breast and/or ovarian Cancer syndromes other than BRCA. J Oncol.

[18] Syngal S, Brand RE, Church JM, Giardiello FM, Hampel HL, Burt RW (2015). ACG clinical guideline: genetic testing and management of hereditary gastrointestinal cancer syndromes. Am J Gastroenterol.

